# Titanium-protein nanocomposites as new biomaterials produced by high-pressure torsion

**DOI:** 10.1038/s41598-022-26716-8

**Published:** 2023-01-10

**Authors:** Ricardo Floriano, Kaveh Edalati, Karina Danielle Pereira, Augusto Ducati Luchessi

**Affiliations:** 1 School of Applied Sciences, University of Campinas (FCA-UNICAMP), Pedro Zaccaria, Limeira, 130013484-350 Brazil; 2 WPI, International Institute for Carbon-Neutral Energy Research (WPI-I2CNER), Kyushu University, Fukuoka, 819-0395 Japan; 3 Institute of Biosciences, São Paulo State University (UNESP), Rio Claro, São Paulo Brazil

**Keywords:** Biomaterials, Composites

## Abstract

The development of new biomaterials with outstanding mechanical properties and high biocompatibility has been a significant challenge in the last decades. Nanocrystalline metals have provided new opportunities in producing high-strength biomaterials, but the biocompatibility of these nanometals needs to be improved. In this study, we introduce metal-protein nanocomposites as high-strength biomaterials with superior biocompatibility. Small proportions of bovine serum albumin (2 and 5 vol%), an abundant protein in the mammalian body, are added to titanium, and two nanocomposites are synthesized using a severe plastic deformation process of high-pressure torsion. These new biomaterials show not only a high hardness similar to nanocrystalline pure titanium but also exhibit better biocompatibility (including cellular metabolic activity, cell cycle parameters and DNA fragmentation profile) compared to nano-titanium. These results introduce a pathway to design new biocompatible composites by employing compounds from the human body.

## Introduction

Biomaterials are receiving considerable attention for different applications in recent years. Developing metallic biomaterials for implants is particularly a critical issue from both research and technical points of view because of the direct contact of the implants with human body tissues, bones and fluids under load. The human body is a very corrosive and complex environment resulting in the occurrence of different corrosion types when a load-bearing artificial material is implanted in the human body^1–3^. The body fluid contains various organic compounds and a notable variety of proteins. There are nearly 105 different proteins available in the human body, each one with a specific role. Among these proteins, albumin was reported to be the most abundant protein in the plasma and synovial fluid^4^, and thus, present in any human tissue where an artificial material could be implanted.

One of the initial stages that significantly influences biocompatibility is the instantaneous adsorption of proteins from biological fluids onto biomaterial surfaces^1,2^. Furthermore, protein adsorption is considered the first and most crucial stage enabling the adhesion of cells on the biomaterial surface and thus relevant clinical phenomena such as osseointegration of orthopedic implants proceed during this stage^1–4^. Albumin was identified as the strongest metal binder among human blood proteins, thus, the adsorption of albumin on implant surfaces plays a key role in determining surface functionalities such as biocompatibility, corrosion and tribology^5^. Proteins create a thick layer on the surface of material and cells sense foreign surfaces through this layer and start to respond. Some reports on implants clearly revealed the presence of protein-containing layers on the surface^1,6^, indicating the importance of the interaction of proteins with biomedical alloys at the cellular level.

Titanium and its alloys have been widely used as potential biomaterials in many different implants because of their low elastic modulus, high fatigue strength, excellent corrosion resistance, better biocompatibility in comparison with other biomaterials such as stainless steels and Co-Cr alloys^7,8^ and low density of 4.5 g/cm^3^ which is about half of stainless steels and Co-Cr alloys^9^. However, the main drawback of titanium and its alloys is their lower strength and hardness compared with stainless steels and Co-Cr alloys^7–9^. Recent studies showed that the nanostructuring of titanium is an effective solution to improve its strength and hardness without deteriorating its biocompatibility^10,11^.

The successful usage of titanium implants depends not only on mechanical properties such as elastic modulus and hardness but also on osseointegration at the bone-implant interface^12^. However, due to the non-bioactivity of Ti-based materials, they cannot bond with bone directly and promote new bone formation on their surface in the early stages of the implantation^13,14^. In order to improve the osseointegration of Ti-based materials, two main methods have been employed based on surface modifications: (1) the control of surface topography with physical and/or chemical change^15,16^; (2) the immobilization of bioactive molecules on the implant surface^17,18^. The second approach, in which coatings rich in proteins such as collagen^19^ and bovine serum albumin (BSA)^5,20–22^ are used, can enhance the biocompatibility of Ti-based alloys.

Several studies^20–24^ demonstrated the beneficial effects on biocompatibility when Ti-based alloys are coated with protein or exposed to solutions with high concentrations of BSA diluted with phosphate-buffered saline (PBS). In Refs.^20–22^, it was shown that coating with BSA inhibits the hydrogen evolution and anodic dissolution reactions, increases the resistance of the protective films, and improves cell adhesion to pure titanium and Ti-based alloys such as Ti-6Al-4V, Ti-6Al-7Nb and Ti-6Al-4V-1Zr (in wt %). In a similar direction, it was also shown that BSA enhances the alloy passive film stability at higher concentrations in the Ti-6Al-4V alloy^23^. In the case of the Ti-3Cu (in wt %) alloy exposed to a solution containing BSA proteins^24^, the corrosion resistance was improved and the antibacterial ability was reduced when the BSA content increased in the solution. However, few studies^25,26^ revealed that the adsorption of protein species such as BSA on the surface of Ti-based alloys can enhance the ion release and hamper the adhesion of cells.

Despite the significance of protein coating in enhancing the biocompatibility of implants, these protein-rich coatings have weak bonding to the metallic substrate. Due to such a weak bonding, delamination can occur over time, leading to the failure of this coating technique for long-term implantation^27,28^. Permanent inclusion of a second phase like proteins into the metallic material can be a potential solution to avoid the delamination problem of protein-coated implants, but there have been no attempts to produce such metal-protein composites.

In this study, to produce biomaterials with high strength and good biocompatibility for long-term implantation, the protein particles are directly inserted as the second phase into pure nanostructured titanium. The bulk nanocomposites are produced by a severe plastic deformation method of high-pressure torsion (HPT). The materials show high hardness and better biocompatibility than pure titanium and do not suffer from delamination problems, which is a general problem of coating with protein. This first introduction of metal-protein composite biomaterials opens a new pathway for a wide range of biomedical applications.

## Materials and methods

### Material synthesis

**Figure 1:**
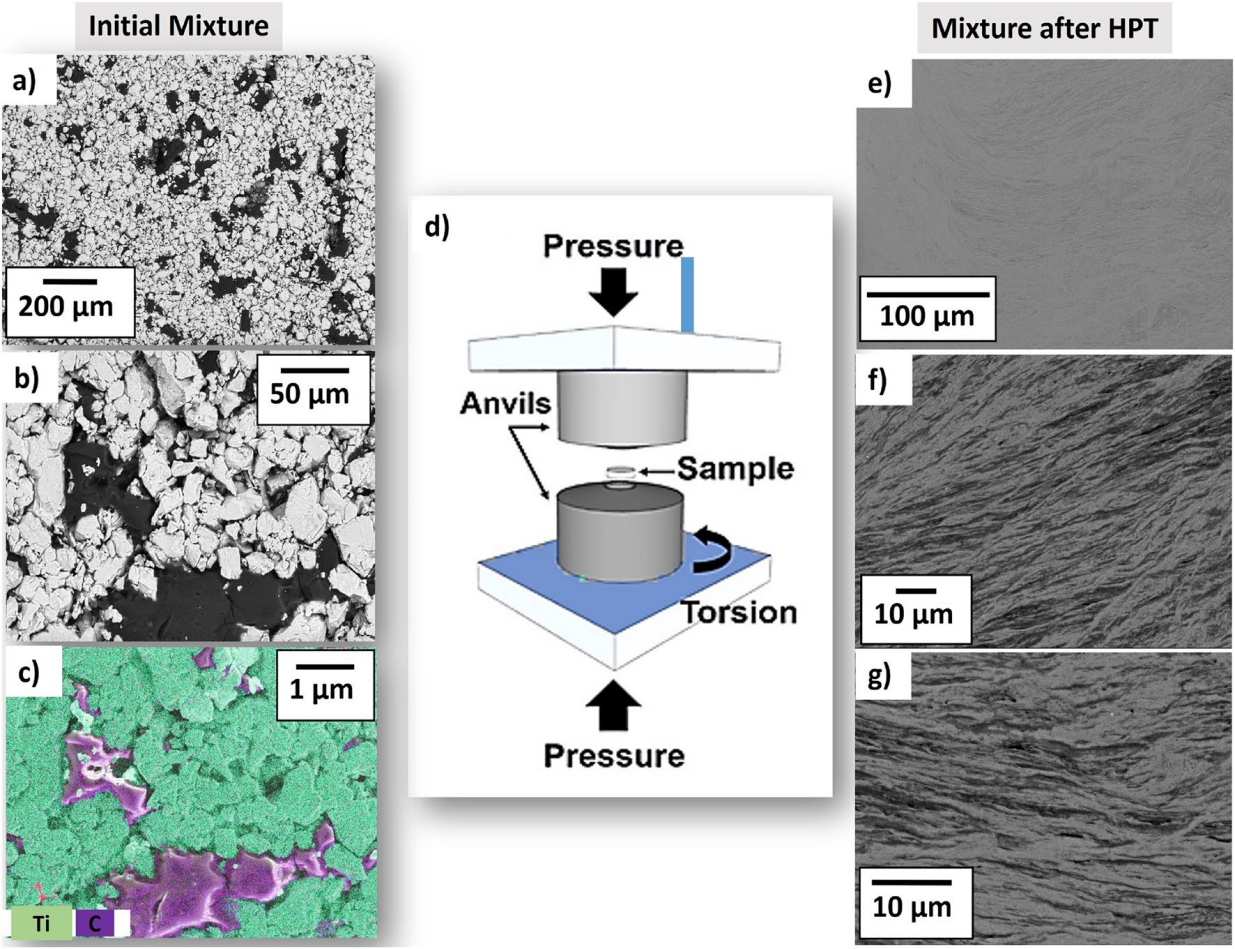
(**a**,**b**) SEM images in BSE mode and (**c**) corresponding EDS elemental mappings of Ti + 5 vol% BSA before HPT processing. (**d**) Schematic of HPT method and its anvils^29^. (**d**,**f**,**g**) SEM images at different magnifications in BSE mode for Ti + 5 vol% of BSA composite after 5 turns of HPT under 2 GPa.

Titanium powder with a purity level of 99.9% and particle sizes below 45 μm and BSA powders were used to prepare the biomaterials. BSA was acquired from Amresco (Solon, OH, USA) with 98% purity and was used without any further purification. The titanium powder was mechanically mixed with BSA particles in the proportions of 0, 2 and 5 vol%. The resulting powder mixtures, as shown in Fig. 1a–c, were then pre-consolidated under an applied pressure of 300 MPa to a disc shape with a diameter of 10 mm and a thickness of ~ 1 mm using a manual hydraulic press. The HPT method was then applied to the compacted discs to prepare the bulk nanocomposites with a good mixing level. The HPT method is a severe plastic deformation technique in which a disc-shaped sample is compressed between two anvils under high pressure and shear strain is induced by rotating one of the anvils with respect to the other one, as shown in Fig. 1d ^29^. The HPT process was conducted under 2 GPa at room temperature for 5 turns with a rotation speed of 1 turn per minute. The HPT method was selected for the synthesis of composites because of three main reasons. (1) This method has been well established as a process capable to produce nanostructured materials with a high density of lattice defects^30–34^. (2) The method can be used to produce bulk composites from powder mixtures at ambient temperature^33,34^. (3) Nanostructured Ti-based materials processed by HPT can show both good biocompatibility and high strength^32–35^.

After HPT processing, the samples were polished to a mirror-like surface for the microstructural, mechanical and biocompatibility investigations. The results of mechanical properties and biocompatibility for the HPT-processed samples were compared with a reference coarse-grained bulk pure titanium (99.9%) with an average grain size of 200 μm which was annealed at 1073 K for 1 h.

### Microstructural and mechanical analyses

Scanning electron microscopy (SEM) technique was used to investigate the microstructure features at the micrometer level. SEM images were taken using the secondary and backscattered electrons (SE and BSE) signals under an acceleration voltage of either 15 kV with a JEOL JSM-7900F microscope or 25 kV with a FEG Philips XL-30 microscope equipped with a Bruker Nano X-Flash 6|60 energy dispersive X-ray spectroscopy (EDS) detector. SEM images were taken at 4 mm away from the center of HPT-processed discs.

Transmission and scanning-transmission electron microscopy (TEM and STEM) were conducted with an acceleration voltage of 200 kV using an aberration-corrected microscope (JEOL JEM-ARM200F). For TEM and STEM, 3 mm diameter discs were cut from 2 to 5 mm away from the center of the HPT-processed disc using an electric discharge machine. The 3 mm discs were first ground to a thickness of 100 µm by abrasive papers and later to a smaller thickness for electron transparency by a conventional twin-jet electrochemical polisher using a solution of 5% HClO_4_, 25% C_3_H_3_(CH_2_)_2_CH_2_OH and 70% CH_3_OH, under a voltage of 12 V at a temperature of 263 K. Microstructural examination by TEM and STEM was conducted by bright- and dark-field (BF and DF) images, high-angle annular dark-field (HAADF) images, selected area electron diffraction (SAED) and EDS mappings.

For crystal structure analysis, the samples were examined by X-ray diffraction (XRD) method using an X’Pert Panalytical diffractometer equipped with a graphite monochromator operating at 45 kV and 40 mA with Cu Kα radiation (wavelength of *λ* = 0.15406 nm).

For mechanical property analysis, Vickers microhardness was measured using a load of 500 gf and a dwell time of 15 s on the upper surface of the samples at four different radial directions from the center to the edges of the HPT-processed discs.

### Biocompatibility tests

The biocompatibility was assessed by two main tests: (1) modified 3-(4,5-dimethylthiazol-2-yl)-2,5-diphenyltetrazolium bromide (MTT) assay; (2) analysis of cell cycle and DNA fragmentation profile by flow cytometry. In the sequence, details of biocompatibility tests are presented.

#### Cell culture and disc plating

Mouse preosteoblast cell lines (MC3T3-E1) were maintained in Alpha Minimum Essential Medium (α-MEM) supplemented with 10% fetal bovine serum (FBS), 2 mM L-glutamine, 100 U/mL penicillin, and 100 μg/mL streptomycin (Life Technologies, Inc., Carlsbad, CA, USA) in a humidified atmosphere containing 5% CO_2_ at 310 K. The MC3T3-E1 cells were provided by the Institute of Biomedical Sciences from the University of São Paulo (USP), São Paulo, Brazil.

MC3T3-E1 cells with an 80% confluence were trypsinized and then inactivated with α-MEM and counted in a Countess II automatic counter (Thermo Fisher Scientific Inc., Walthan, MA, USA). The HPT-processed discs were arranged in 24-well plate (1 disc per well). The discs were sterilized by overnight exposure to ultraviolet light in a biosafety cabinet and 60 µL of cellular suspension (1 × 10^5^ cells) were plated on the surface of the discs. The plate was incubated in a humidified atmosphere containing 5% CO_2_ at 310 K for a period of 2 h. Then, 1 mL of α-MEM was added to each well and the plate was incubated in a humidified atmosphere containing 5% CO_2_ at 310 K.

#### MTT assay

MTT assay is used to measure cellular metabolic activity as an indicator of cell viability, proliferation and cytotoxicity. The MTT assay is based on the reduction of a yellow tetrazolium salt, 3-(4,5-dimethylthiazol-2-yl)-2,5-diphenyltetrazolium bromide (MTT) to purple formazan crystals by metabolically active cells. After 48 h of plating, the culture media were aspirated, and MTT (0.5 mg/mL in PBS) was added to cells and then incubated for 3 h in a humidified atmosphere containing 5% CO_2_ at 310 K. The growth media was discharged and 250 μL of dimethyl sulfoxide (DMSO) was added to each well to dissolve MTT. Light absorbance was determined at 570 nm using a scanning spectrophotometric multiwell plate reader (F5 Microplate Reader, Molecular Probes).

#### Analysis of cell cycle and DNA fragmentation profile by flow cytometry

For the flow cytometry assays, the HPT-processed discs were placed in a 24-well plate (1 disc per well), sterilized in ultraviolet light for 12 h and rinsed in phosphate-buffered saline. About 1.8 × 10^5^ cells were plated in each disc. After 48 h of plating, cells were harvested and washed with PBS. Cold ethanol (70%) was used to fix cells for 30 min and 50 μg/mL propidium iodide (PI) diluted in PBS containing 1 mg/mL RNase was used to stain for 30 min. The cells were maintained at room temperature in the dark. Percentages of cells in different phases of the cycle were evaluated by flow cytometric analysis based on PI-stained nuclei using Accuri™ C6 software (BD Biosciences).

## Results

### Microstructural characterization

Figure 1a–c show SEM images including the EDS elemental mapping of selected elements of the compact disc before HPT processing for composite containing 5 vol% of BSA. In this case, the surface of this sample did not receive any polishing procedure. As can be seen by the composition contrast in the BSE images (Fig. 1a,b), there is a reasonable mixing between the titanium and BSA particles at the micrometer level. The gray particles refer to titanium with dimensions ranging from 10 to 45 μm while the black particles refer to the BSA protein. BSA is a linear polymer containing C, H, O, N and S atoms^4^. The elemental mapping shown in Fig. 1c confirms that the black particles correspond to the BSA protein containing carbon atoms which are surrounded by a titanium matrix.

Figure 1e–g show BSE images for the composite containing 5 vol% of BSA after HPT processing. As indicated by the composition contrast and EDS analysis, the black regions are related to the BSA protein. These images show that after severe plastic deformation carried out by HPT processing, the BSA protein is mixed with titanium particles forming well-defined protein layers which are distributed all over the sample, while the titanium phase keeps its three-dimensional network. The capability of HPT to form a layered structure of titanium and BSA is a consequence of the shear strain effect on the ballistic microstructural evolution of titanium and BSA, while high hydrostatic pressure facilitates the co-deformation of two components with different structures and properties. This capability of the HPT method was already employed to produce various Ti-based composites such as metal–metal composites^36^, metal-ceramic composites^37^, and metal–carbon composites^38^. Here, it should be noted that the microstructures shown in Fig. 1e–g were also observed when the Ti-BSA composite was examined by SEM from the cross-sectional view, a feature that is usually observed in HPT-processed metals^30^.

**Figure 2:**
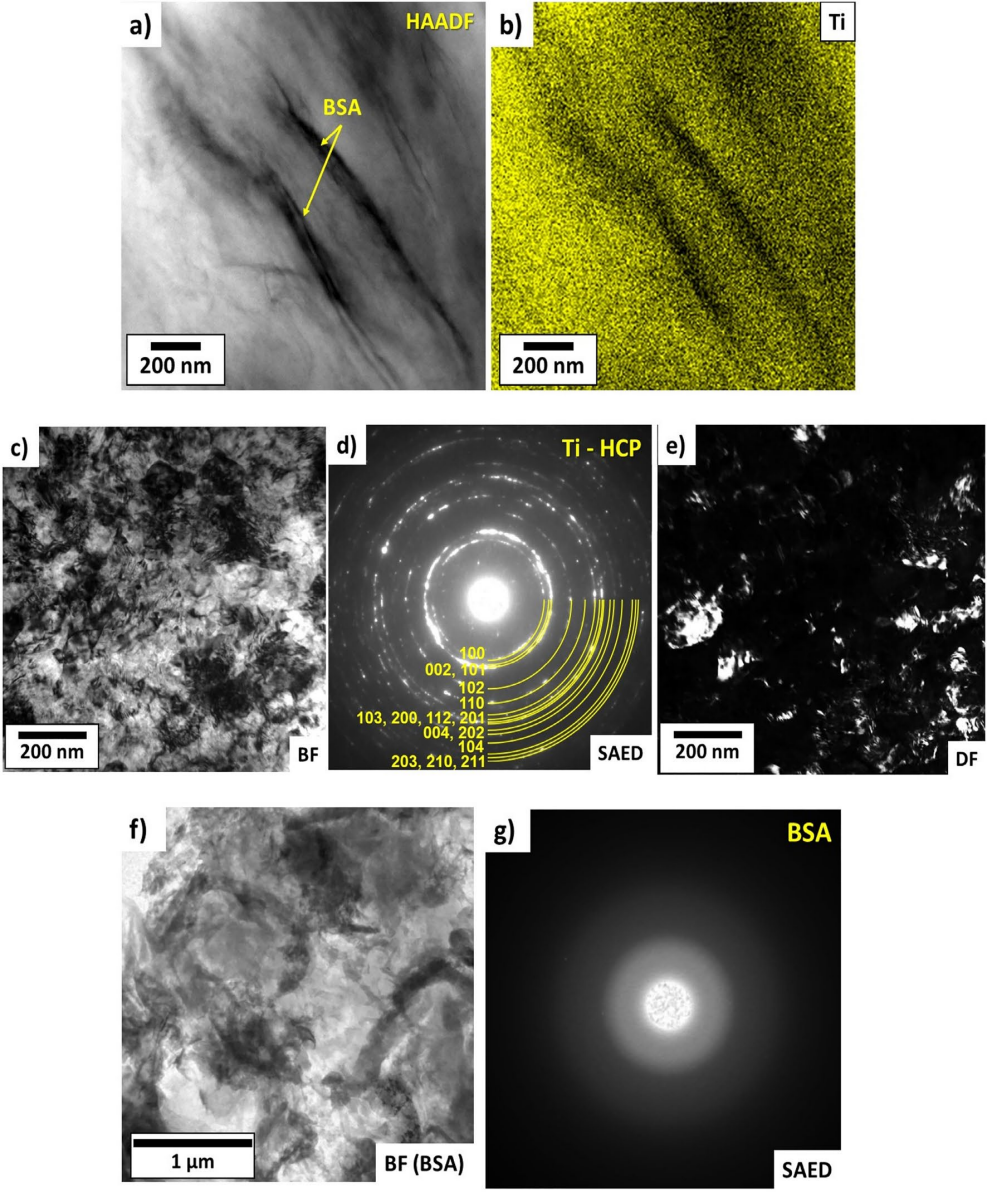
STEM and TEM analyses for Ti + 5 vol % BSA composite after 5 turns of HPT under 2 GPa. (**a**) HAADF image and (**b**) and corresponding EDS mapping with Ti; (**c**,**f**) TEM-BF images; (**d**,**g**) SAED patterns corresponding to (**c**,**f**); and (**c**) TEM-DF image.

Examinations of microstructure in a higher magnification using STEM-HAADF and EDS, as shown in Fig. 2a,b, confirm that titanium and BSA have a good bonding at the nanometer scale. Although future theoretical calculations are needed to clarify the nature of this bonding, their features should be similar to those observed for the absorption of protein on titanium implants in the human body. The bonding between titanium and protein generally follows two steps: (1) hydrogen bonding, and (2) proton transfer^39^. Some studies suggested that such bonding is enhanced by the interaction of the OH group with titanium^40,41^, while molecular dynamics simulations reported the significance of the electrostatic interactions of the amide and carboxyl groups on the bonding^42,43^.

TEM analysis was employed to explore more features of the microstructure of composite at the submicrometer and nanometer level. Figure 2 shows representative TEM images for the composite with 5 vol% of BSA after HPT processing. The BF and DF images (Fig. 2c,e) show the presence of several titanium ultrafine grains with nanometer or submicrometer sizes with an average grain size of 90 nm. The SAED pattern (Fig. 2d) taken from the region shown in Fig. 2c shows well-defined rings, suggesting the presence of ultrafine grains with random orientations in the selected region. The apparent rings in Fig. 2d belong to the titanium with the hexagonal close-packed (HCP) structure. A BF image (Fig. 2f) together with the respective SAED pattern (Fig. 2g), taken from a region of the composite containing the BSA protein, show the amorphous nature of BSA which is well characterized by the halo ring pattern shown in the SAED pattern.

**Figure 3:**
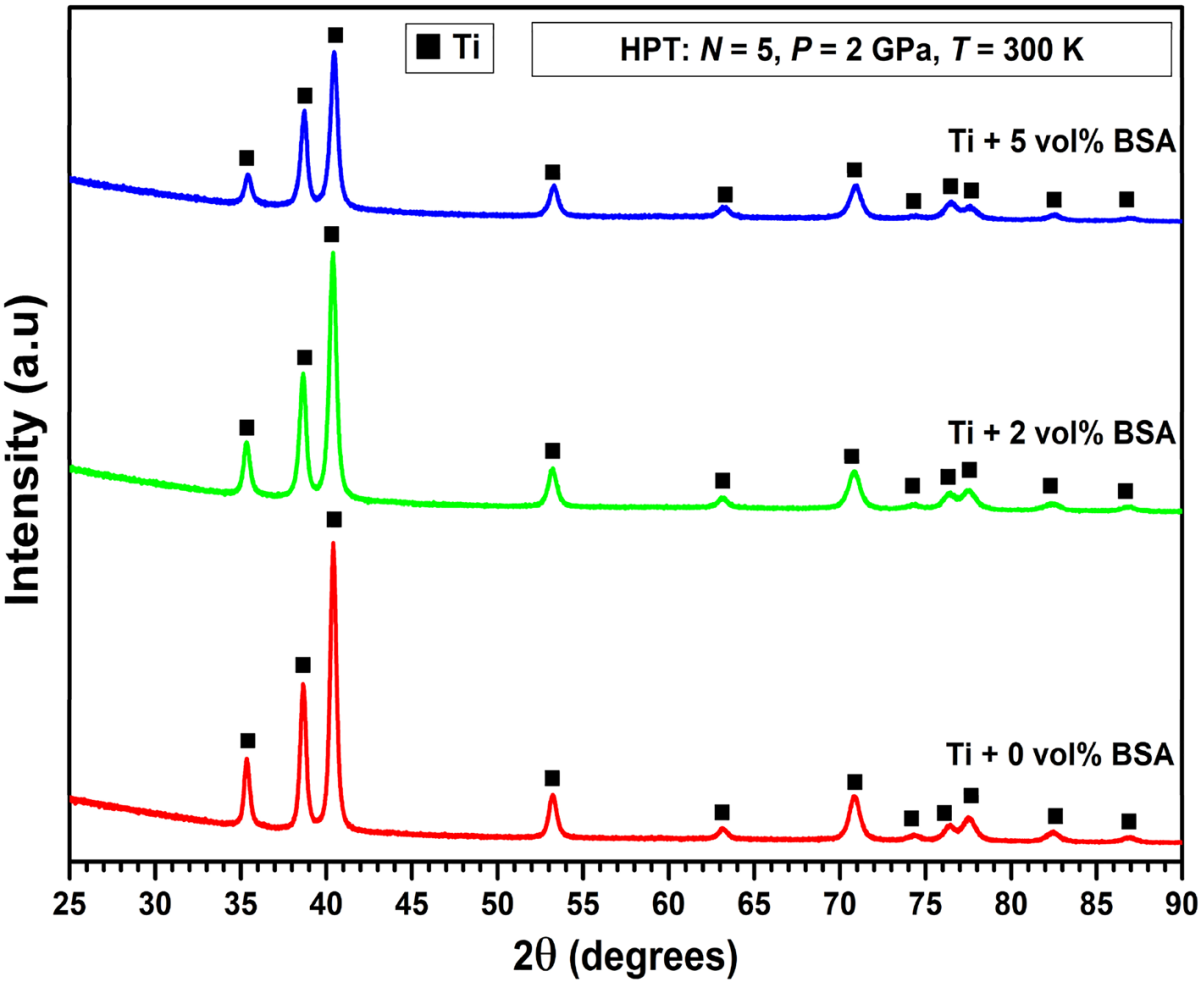
XRD patterns for pure titanium and for the composites containing 2 and 5 vol% of BSA after 5 turns of HPT under 2 GPa.

Figure 3 shows the XRD patterns for pure titanium and for the samples containing 2 and 5 vol% of BSA after processing with HPT. As can be well noticed, the XRD patterns of all samples show the presence of a single phase which refers to the titanium hexagonal structure (*P63/mmc*) in good agreement with the TEM analysis. No new phases appear in the XRD patterns after HPT processing. The only difference between the XRD patterns is related to the decrease of intensities of XRD peaks with the increase of BSA proportion.

### Mechanical characterization

**Figure 4:**
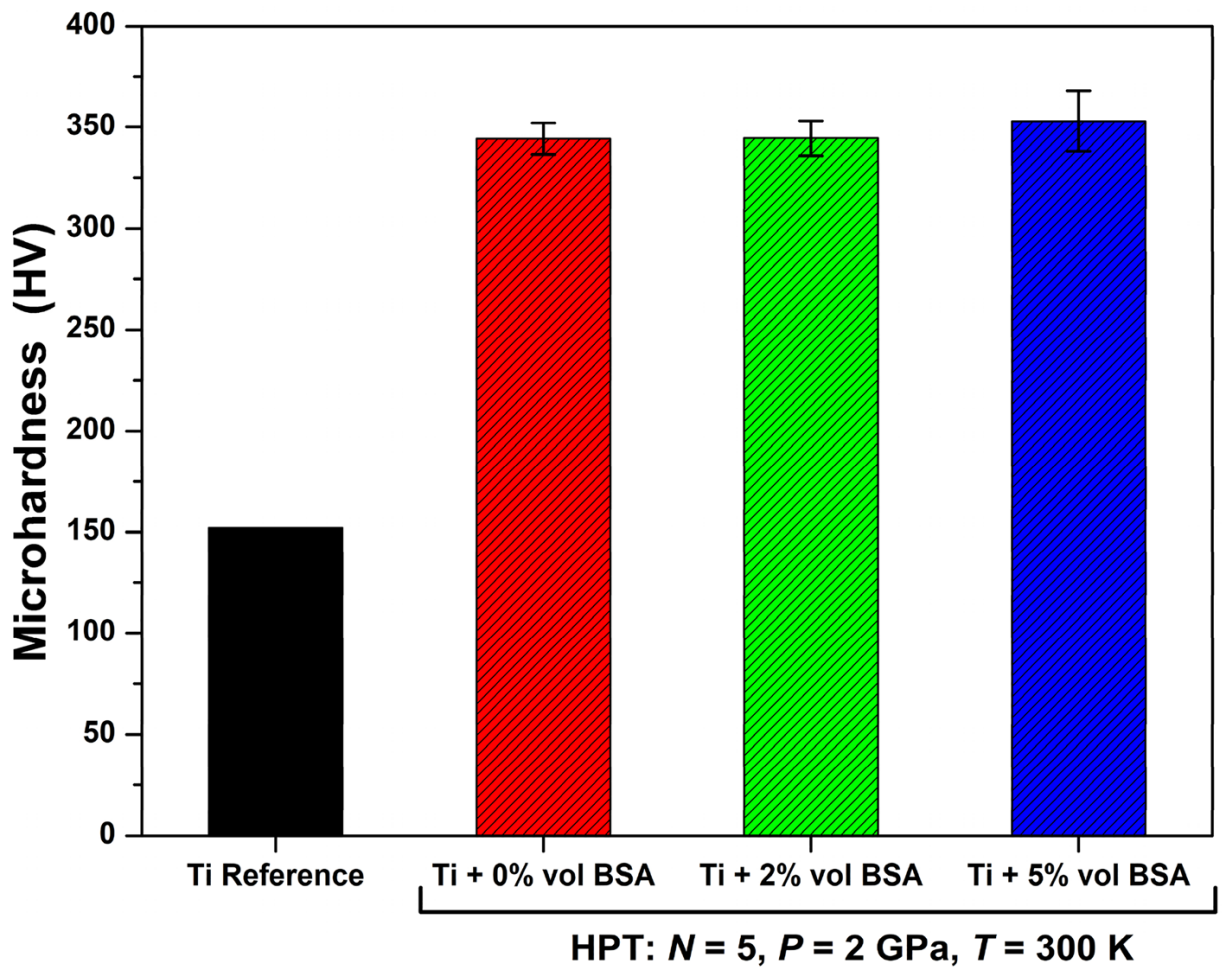
Vickers Microhardness for pure titanium and for nanocomposites containing 2 and 5 vol% of BSA produced by 5 turns of HPT under 2 GPa in comparison with hardness of coarse-grained annealed titanium.

To have an idea about the mechanical properties of samples after the addition of BSA protein by HPT processing, Vickers microhardness obtained at 4 mm away from the center of the discs, where shear strain is maximum, was compared. Figure 4 displays the average values of microhardness for the samples after HPT processing. The values obtained for microhardness are quite similar among the three HPT-processed samples showing that the small additions of BSA up to 5 vol% do not negatively influence the hardness. The measured values are in the range of 344 to 353 HV which is in good agreement with the values found in the literature for Ti-based alloys processed by HPT^34,44^. Figure 4 shows that the hardness of the Ti-BSA composites is over two times higher than the hardness of reference coarse-grained annealed titanium.

Here, two points should be mentioned regarding the mechanical properties. First, hardness was the lowest at the center of discs but increased with increasing the distance from the disc center, as shown in Fig. S1 of Supporting Information. These heterogeneities, which are due to an increase in the shear strain with increasing the distance from the disc center, can be avoided by increasing the number of turns so that hardness values from the disc center to the edge saturate to the steady states^29,30^. Second, while consolidation of pure titanium powders by HPT resulted in 1 GPa tensile yield strength and 12% ductility^45^, the presence of the protein phase resulted in no ductility under tension, a fact that is usually observed in metal-ceramic composites. Despite the good mechanical performance of composites under compressive loads, their limited ductility under tension should be always considered when they are used for any applications including biomedical applications^46^.

### Biocompatibility tests

**Figure 5:**
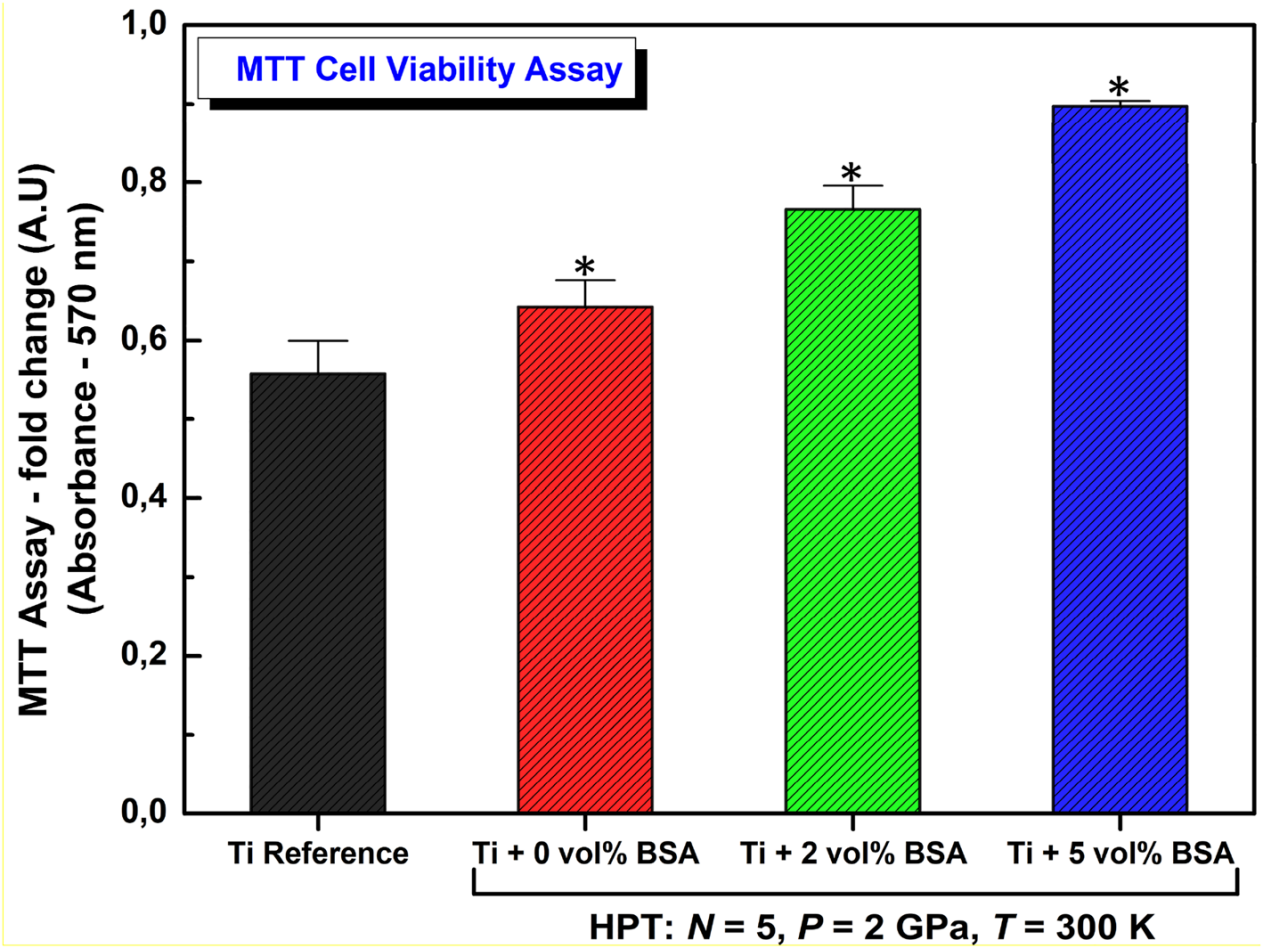
MTT cell viability assay examined by light absorbance at 570 nm for pure titanium and for nanocomposites containing 2 and 5 Vol% of BSA produced by 5 turns of HPT under 2 GPa in comparison with hardness of coarse-grained annealed titanium. Data are shown as mean ± standard deviation of experiments performed in quadruplicate and compared by Kruskal–Wallis ANOVA and pairwise comparisons by Mann–Whitney test.

The cellular viability of the nanocomposites after HPT processing was evaluated by in vitro cell culture experiments employing the MC3T3-E1 cells. The results of the biocompatibility assessment via the MTT assay are shown in Fig. 5. It should be noted that higher light absorbance in Fig. 5 indicates higher cellular metabolic activity. Firstly, as can clearly be seen in Fig. 5, all samples processed by HPT present superior biocompatibility in comparison with the coarse-grained titanium reference. In a similar direction, Refs^32,34,35^ also showed the improvement of biocompatibility in Ti-based alloys after HPT processing. Furthermore, among the samples processed by HPT in Fig. 5, the samples containing BSA showed superior biocompatibility in comparison to those samples without BSA. The improvement in biocompatibility in the HPT-processed samples seems to be proportional to the BSA content and the sample with 5 vol% of BSA shows the best biocompatibility behavior. Since the adsorbed proteins in the surface of the implant mediate the cell-surface interactions including its differentiation and proliferation, it is normally expected that a better osseointegration process and consequently an excellent tissue integration achieved on the Ti-BSA nanocomposites, especially in the early stages of implantation.

**Figure 6:**
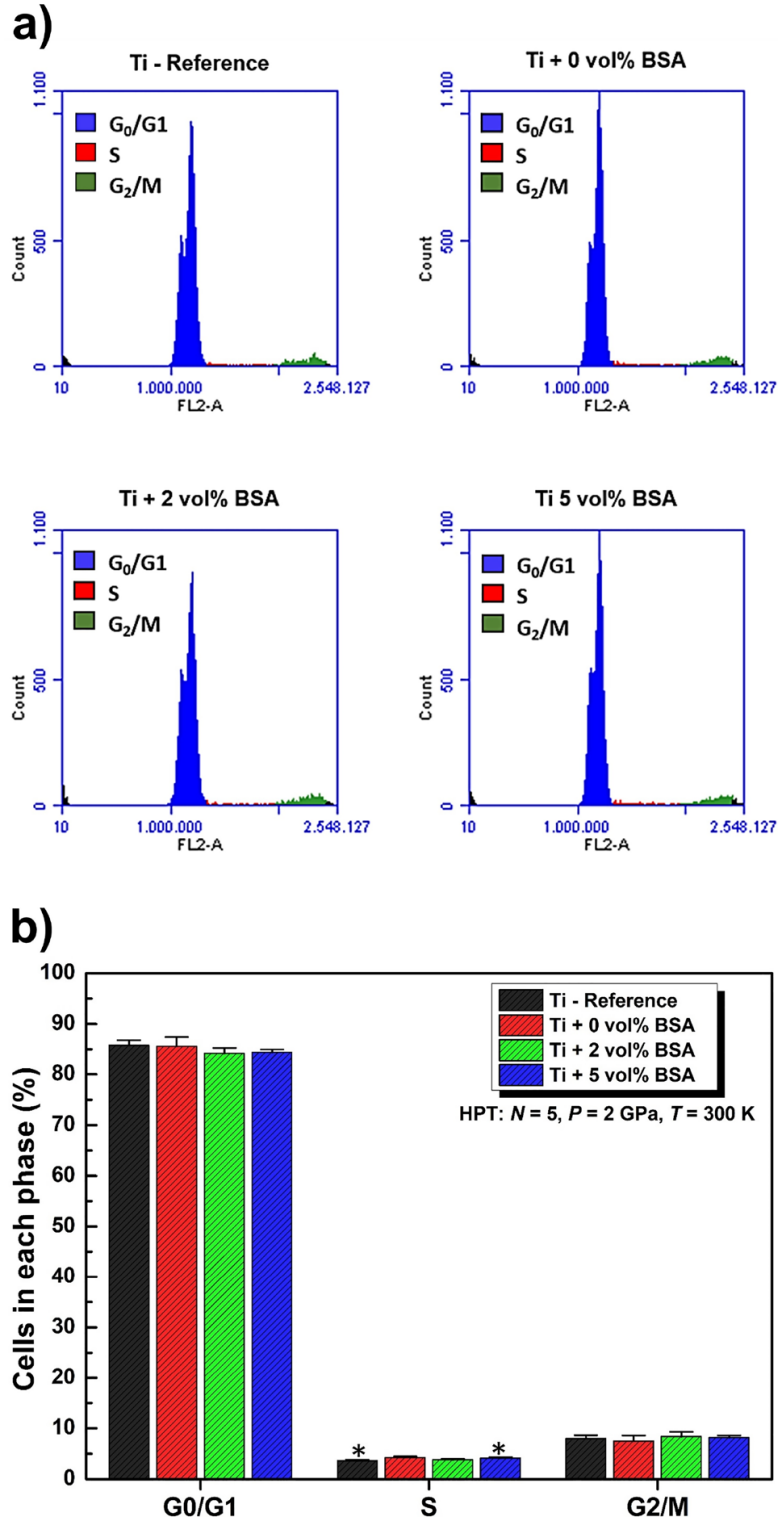
Cell cycle profile of MC3T3-E1 cells. (**a**) Representative histogram for the samples showing distribution of cells in the individual phases of cell cycle in the G0/G1 (Blue), S (Red), and G2/M (Green) phases. (**b**) Quantitative analysis of distribution or proportion of the cell numbers in each phase, performed from at least 10,000 events per sample. Data are shown as mean ± standard deviation of experiments performed in quadruplicate and compared by Kruskal–Wallis ANOVA and pairwise comparisons by Mann–Whitney test.

Figure 6 shows the cell cycle profile of MC3T3-E1 cells for the samples processed by HPT including a titanium reference sample. The cells were stained with propidium iodide (PI), the emitted fluorescence served as a pulse signal, and the signal area (FL2-A) was determined. For cell cycle analysis, 10,000 events were collected on the histogram plot of FL2-A. In Fig. 6a, PI data is on a histogram with the cell count on the y-axis and the PI fluorescence intensity on the x-axis. The histogram shows the number of cells in three different phases: G0/G1; S and G2/M for 48 h. As can be observed in Fig. 6b, the number of cells presented in G0/G1, S and G2/M phases, is quite similar among all samples indicating that the addition of BSA did not promote any distortion or anomaly such as increasing or decreasing the number of cells in different phases of cycle profile. In other words, the number of cells in each phase is kept unaltered during the cycle profile independent of the composition or processing route.

**Figure 7:**
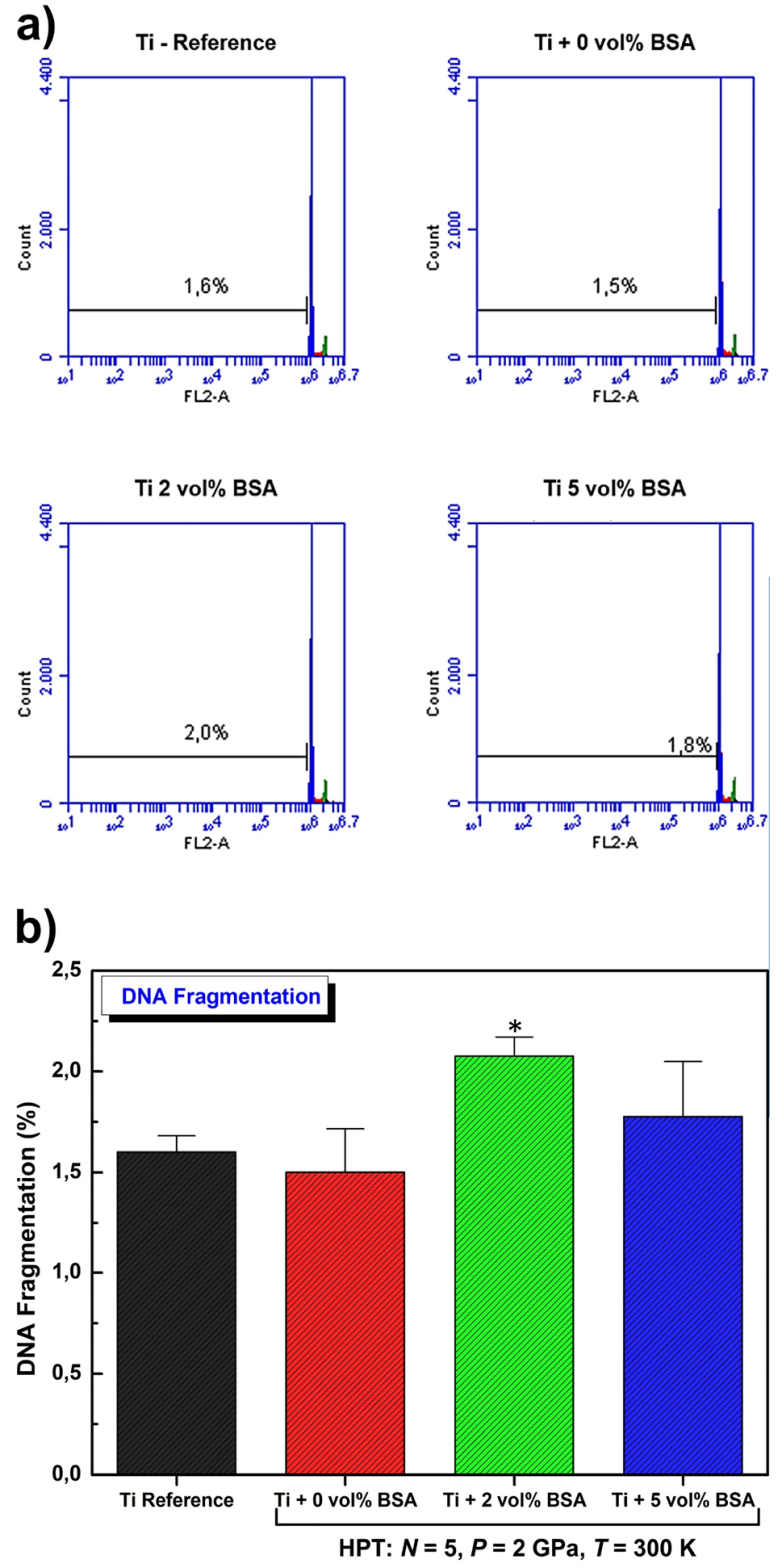
DNA fragmentation of MC3T3-E1 cells. (**a**) Representative histograms of percentage of cell fragmentation. (**b**) The number of cells with fragmented DNA is shown as the percentage. Data are shown as mean ± standard deviation of experiments performed in quadruplicate and compared by Kruskal–Wallis ANOVA and pairwise comparisons by Mann–Whitney test.

Figure 7 displays the DNA fragmentation of MC3T3-E1 cells for the samples processed by HPT including a titanium reference. In Fig. 7a, the histogram of cellular DNA fragmentation reveals that the samples present values in the range of 1.5 to 2.0% for DNA fragmentation. This indicates that the BSA addition or even the HPT processing does not induce an unexpected behavior in the samples, especially because, the values showed in Fig. 7b are inside of a very narrow range to support any significant changes. Therefore, similar to the conclusion taken from the cell cycle analysis, the addition of BSA to titanium does not induce apoptosis in MC3T3-E1 cells by increasing DNA fragmentation after being processed by HPT.

## Discussion

In the present investigation, metal-protein nanocomposites with high strength and good biocompatibility were introduced for long-term implantation. The nanocomposites are mixtures of a biocompatible metal such as titanium and small amounts of an endogenous protein such as 2 and 5 vol% of BSA. The HPT method was selected to synthesize these new composites because the method ensures a good level of mixture at the nanometer level between the two phases, and it attains the desirable nanostructure with high hardness for biomedical applications.

The initial characterization of the metal-protein nanocomposites performed by SEM, STEM, TEM and XRD analyses revealed interesting microstructural aspects of these new biomaterials. The composites showed a good level of mixing between titanium and BSA protein together with the presence of a nanocrystalline structure containing ultrafine grains of titanium with an average grain size of 90 nm. The production of ultrafine grains and nanocrystalline materials which exhibit enhanced hardness is one of the most attractive aspects of HPT for biomaterial applications^29–35^. No new phases were identified in the metal-protein nanocomposites after the HPT process, which indicates that the high hardness is due to nanostructuring and not due to the ω-Ti phase formation^45^. Due to these microstructural features, the metal-protein nanocomposites showed hardness levels two times higher than the hardness of reference coarse-grained titanium. Moreover, the BSA addition (up to 5 vol% of BSA) did not negatively influence the hardness, confirming that protein addition in small amounts is a practical solution to develop high-strength biomaterials.

The biocompatibility of metal-protein nanocomposites after HPT processing was tested by direct cell culture experiments employing the MC3T3-E1 cells. The results showed that the metal-protein nanocomposite containing 2 and 5 vol% of BSA presented superior biocompatibility in comparison with the pure nanocrystalline and coarse-grained titanium references. Beyond that, the composite containing 5 vol% of BSA showed the best biocompatibility behavior among all samples. Other important aspects related to the interaction of cells with the metal-protein nanocomposite were also shown in the cell cycle profile and DNA fragmentation results. Those tests indicated clear pieces of evidence that the addition of BSA to titanium did not induce any distortion or anomaly such as increasing or decreasing the number of cells in different phases of the cycle profile and also did not induce the apoptosis in MC3T3-E1 cells by increasing the DNA fragmentation after being processed by HPT. The superior biocompatibility of the metal-protein nanocomposites can be attributed to the presence of BSA proteins in the whole material including their surfaces that interact with the MC3T3-E1 cells showing excellent cell proliferation and adhesion. It should be noted that although temperature rise during HPT is of minor significance to lead to thermal denaturation^47,48^, cold denaturation and protein unfolding process of BSA can occur under high pressure of 2 GPa, as suggested in earlier publications^49,50^. Despite these possible three-dimensional structural changes under high pressure, the BSA remains effective to enhance biocompatibility in current Ti-BSA composites.

This positive biocompatibility effect due to the presence of BSA in Ti-based alloys is in good agreement with the reported effect of protein coating on implants^20–24^. While protein-coated implants suffer from delamination for a long time, the BSA protein was introduced as a second phase in titanium by cold consolidation via the HPT process in this study. Therefore, this new family of metal-protein nanocomposites is expected to be much less prone to the delamination problem, although long-term tests are required to confirm this issue. Because cold consolidation by HPT is applicable to almost any kinds of composites, this study opens a pathway for the design and synthesis of a wide range of metal-protein nanocomposite biomaterials.

## Conclusions

In this study, we introduce metal-protein nanocomposites as new biomaterials with high hardness and excellent biocompatibility. The nanocomposites were synthesized by mixing pure metallic titanium with bovine serum albumin (BSA), an endogenous mammalian protein, by high-pressure torsion. The following conclusions were drawn from this study.

- Electron microscopy showed a good level of mixing between titanium and BSA protein, while the grain size of titanium was well at the nanometer level.
- The microhardness of metal-protein nanocomposites was similar to nanocrystalline pure titanium and over two times higher than coarse-grained pure titanium.
- The X-ray diffraction showed that the addition of BSA and subsequent processing did not promote the formation of new phases into the material.
- The cellular viability of samples, evaluated by in vitro cell culture experiments using the MC3T3-E1 cells, showed that the addition of 5 vol% of BSA results in the best biocompatibility due to enhanced cell proliferation promoted by the presence of BSA biomolecules.
- The addition of BSA did not promote any alteration in the cell profile and DNA fragmentation, indicating that the biomaterial studied here would be an excellent option to be used as an implant with high hardness and high biocompatibility.

## Supplementary Information

Supplementary Figure S1.

## Data Availability

The datasets generated during and/or analysed during the current study are available from the corresponding author on reasonable request.
