# Supplementary material for: Titanium-protein nanocomposites as new biomaterials produced by high-pressure torsion
Source: Sci Rep. 2023 Jan 10;13:470. doi: 10.1038/s41598-022-26716-8 (PMC9832118; doi:10.1038/s41598-022-26716-8)
Supplement: Supplementary file 1 — Supplementary Figure S1. [file 41598_2022_26716_MOESM1_ESM.docx]

**Supporting Information**

**Titanium-Protein Nanocomposites as New Biomaterials Produced by**

**High-Pressure Torsion**

Ricardo Floriano^1,*^; Kaveh Edalati^2^; Karina Danielle Pereira^1^; Augusto Ducati Luchessi^1,3^

^1^ School of Applied Sciences, University of Campinas (FCA-UNICAMP), Pedro Zaccaria, 1300, 13484-350, Limeira, Brazil

^2^ WPI, International Institute for Carbon-Neutral Energy Research (WPI-I2CNER), Kyushu University, Fukuoka 819-0395, Japan

^3^ Institute of Biosciences, São Paulo State University (UNESP), Rio Claro, São Paulo, Brazil.

***Corresponding author:**

Ricardo Folriano (E-mail: rflorian@unicamp.br)

**Figure S1** shows that the Vickers microhardness increases with increasing the distance from the disc center due to increasing shear strain. While the hardness of pure titanium reasonably saturates to a steady state at radiad distances of larger than 1 mm, the hardness for the composites containing 2 and 5 vol% of BSA do not reach the steady states after 5 turns of HPT. Therefore, larger numbers of HPT turns (i.e. larger shear strains) should be applied in future studies to achieve a steady-state microstructure and constant hardness in the Ti-BSA composite discs. Despite the difference in the evolution of hardness with the shear strain of the three samples, their hardness is quite similar at 4 mm away from the disc center, where the applied shear strain is maximum.


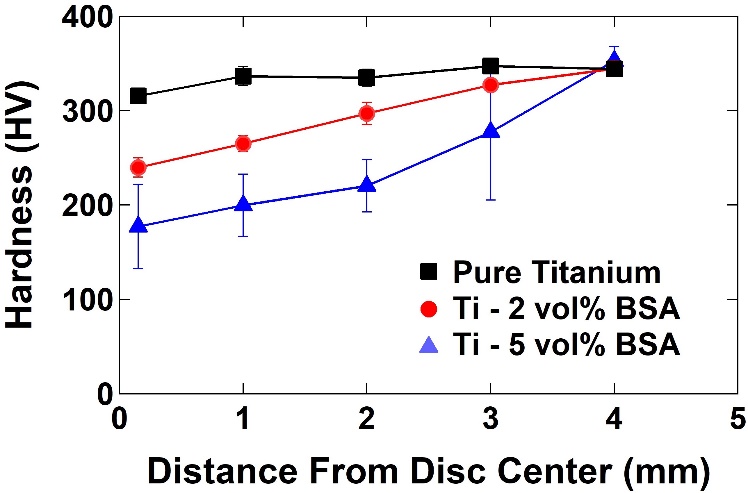


**Figure S1.** Variations of hardness versus distance from disc center for pure titanium and for the composites containing 2 and 5 vol% of BSA after 5 turns of HPT under 2 GPa.
